# Non-Destructive Assessment of Aroma Volatiles from a Climacteric Near-Isogenic Line of Melon Obtained by Headspace Stir-Bar Sorptive Extraction

**DOI:** 10.3390/foods2030401

**Published:** 2013-08-28

**Authors:** Juan Pablo Fernández-Trujillo, Noelia Dos-Santos, Rocío Martínez-Alcaraz, Inés Le Bleis

**Affiliations:** Department of Agricultural and Food Engineering, Technical University of Cartagena (UPCT), Paseo Alfonso XIII, 48, ETSIA & Institute of Plant Biotechnology, E-30203 Cartagena (Murcia), Spain; E-Mails: noelia.dossantos@upct.es (N.D.-S.); rociomartinez_65@hotmail.com (R.M.-A.); ines.lebleis@neuf.fr (I.L.B.)

**Keywords:** climacteric ripening, *Cucumis melo* L., ethylene, introgression lines, fruit quality, HSSE, postharvest

## Abstract

A climacteric aromatic near-isogenic line (NIL) of melon (*Cucumis melo* L.) SC3-5-1 contained an introgression of the non-climacteric Korean cultivar “Shongwan Charmi” accession PI 161375 (SC) in the genetic background of the non-climacteric cultivar “Piel de Sapo” (PS). The aroma production was monitored during ripening at 21 °C in intact fruit using headspace sorptive bar extraction (HSSE). Bars were composed of polydimethylsiloxane (PDMS) and aromas were desorbed and analyzed by gas-chromatography mass-spectrometry. The aromatic profile was composed of 70 aromatic compounds plus 21 alkanes with a predominance of esters, particularly acetate (2-methylbutyl acetate, 2-methylpropyl acetate, hexyl acetate, and phenylmethyl acetate). Some compounds were severely affected by postharvest time. The acetate esters (3-methylbutyl acetate, butan-2-yl acetate and phenylmethyl acetate) decreased with ripening and sulfur-derived compounds (*S*-methyl butanethioate and *S*-methyl 3-methylbutanethioate) increased gradually with ripening. A few compounds increased at the senescence phase (propyl ethanoate). Other compounds such as hexadecanoic acid showed a marked decrease after harvest, some decreasing from a relative maximum at harvest (2-methylpropyl hexanoate; *n*-hexanoic acid; nonanoic acid).

## 1. Introduction

Climacteric or non-climacteric behavior is an interesting topic in fruits ripening with potential implications for insect attraction, seed dispersal and readiness for predation, or human consumption [1]. The production of volatiles associated with climacteric behavior is common to many fruit but only a few of them such as melons or plums have cultivars showing a differential behavior that permits the in-depth study of these traits [2,3,4,5,6]. Based on differences in the intensity of climacteric behavior, Obando* et al.* [7] proposed at least two QTLs controlling this character, one at least (*eth3.5*) previously mapped in LG III [8,9]. 

The climacteric behavior of this NIL is strongly associated with a typical aromatic profile [3] and softening associated with cell wall degradation and accelerated ripening compared with the non-climacteric inbred “Piel de Sapo” parental [10,11,12]. Recently, Vegas *et al.* [12] showed that SC3-5-1, a climacteric NIL of melon, have two introgressions in melon linkage groups III and VI, respectively, of the Korean accession PI 161375 in a “Piel de Sapo” genetic background. In intact fruit of SC3-5-1 harvested close to climacteric peak, Fernández-Trujillo *et al.* [13] showed an increase of total acetate esters, and a sudden decrease in alcohols, accompanied by an upsurge in non-acetate esters and maximum ethylene production lasting around 3 days. However, the main individual aroma volatiles of NIL SC3-5-1 fruit during ripening have not been reported. 

The goal of this paper is to characterize the main individual changes in volatiles associated with SC3-5-1 climacteric fruit ripening and senescence, particularly those revealing potential ethylene-dependent behavior in intact fruit after harvest.

## 2. Experimental Section

Fruits obtained from melon (*Cucumis melo* L.) plants of the NIL SC3-5-1 were harvested in full-ripe stage of maturity in mid July 2009 in Cartagena, Murcia, SE Spain. Harvest indices, experimental design and flesh sampling followed the methodology reported by [7]. The inbred parents showed non-climacteric behavior, while SC3-5-1 is a climacteric NIL [9,13].

For ripening experiments, fruit were stored at 21 ± 1 °C and 93% ± 3% RH for 10 days. For aroma volatile analysis, four selected fruits (one fruit per replicate) harvested at the end of the season for SC3-5-1 were used. Fruit weight and density (mean ± SE, *n* = 4) were 1636 ± 88 g and 992 kg·m^−3^, respectively. All the fruit samples were sampled for 1 h within hermetic containers of 5283 cm^3^ at harvest and during ripening.

A method previously reported [8] was used for sampling carbon dioxide (after 1 h) and ethylene (after 45 min) with two 1-mL syringes in order to monitor respiration rates and ethylene production after injecting 0.5 mL of the headspace collected into different gas chromatographs.

For sampling aroma volatiles non-destructively, the Gerstel twister used was of 0.5 mm thickness, 10 mm length, 24 µL volume of polydimethylsiloxane (PDMS; Gertsel GmbH, Mülheim an der Ruhr, Germany). The bars were stacked onto the metallic wall of the container for absorbing aroma headspace. Also, they followed the conditioning process before or after analysis previously reported [13]. The Twister aroma automatically entered the thermal desorption unit (TDU) to be splitlessly desorbed, but with high desorption flow, into the liner (for Gerstel CIS4/Twister desorption unit filled with deactivated quartz wool) of the programmable temperature vaporizing inlet (PTV), where the analytes were cryogenically trapped before detection of the organic compounds adsorbed on the PDMS coating by GC-MS.

Volatile analysis was performed as in [13] on a 6890 gas chromatograph (Agilent Technologies, Palo Alto, CA, USA) equipped with a Gerstel cooled injection system (CIS4 PTV injector) and a Gerstel MultiPurpose Sampler (MPS2) with the Gerstel twister^®^ Desorption Unit (TDU) option and a mass spectrometer 5975 with an hyperbolic quadrupole (Agilent Technologies). For the TDU, the following parameters were used: for the desorption program, 40 to 250 °C (5 min) at 300 °C·min^−1^; carrier gas (He) flow rate, 45 mL·min^−1^. The TDU settings were splitless mode with a fixed transfer temperature of 300 °C and standard sample mode. The back inlet (CIS4) worked in solvent vent mode at initial temperature of 250 °C, 8.60 psi (around 59.3 kPa) pressure, a vent flow of 50 mL·min^−1^, vent pressure of 7.25 psi (around 49.99 kPa), purge flow of 9.2 mL·min^−1^ and total flow of 13.3 mL·min^−1^.

The PTV was cooled to −100 °C with an equilibration time of 0.5 min using liquid nitrogen, the GC cool down time being 0.1 min with a cryogenic timeout of 30 min (cryogenic cooling parameters). The cryogenic cooling temperature program was as follows: injection temperature 250 °C, reached at 10 °C·s^−1^. The hold time was 3 min.

Capillary GC-MS analyses were performed using an HP-5MS ultra inert (Agilent Technologies) column (0.25 mm × 30 m length × 0.25 µm). Chromatographic conditions were as follows: solvent delay, 0.10 min; split mode 10:1; oven temperature program with an equilibration time of 0.50 min and a ramp from 60 to 240 °C at 3 °C·min^−1^. The column operated at constant pressure mode using pentadecane (Sigma-Aldrich-Fluka, Germany) to block the method at 27.5 min [13].

The chromatograms and mass spectra were evaluated using ChemStation software (G1701DA D.02.00.275, Agilent Technologies). The peaks were registered using a mass spectrometer (5973 Network Mass Selective Detector, Agilent Technologies) coupled to the GC. Volatile compounds were tentatively identified by comparing the experimental spectra with those of the National Institute for Standards and Technology (NIST05a.L) data bank [3]. The compounds with a match quality (MQ) higher than 80% in the NIST database were considered for the aroma profile and the rest of the areas were discarded. In order to suppress compounds not associated with melon aroma, a thorough literature and internet search was also performed to determine the identities of these compounds. Linear retention indices mostly reported in the NIST database or in literature searches for HP-5, DB-5 or similar columns were also used to confirm these compounds. Levels of volatile compounds were expressed as a percentage of the total area counts recorded in each chromatogram and the data were then averaged.

Raw data or data transformed into their respective logarithm were analyzed by analysis of variance of repeated measurements with ripening time as fixed factor. When time was significant, mean differences were separated by LSD test with type-I error α ≤ 0.05. Only compounds showing a significant effect of the ripening time are reported as time-dependent and for the rest only the mean ± SE is reported. The rest of compounds of the profile with a presence of at least 47% in the samples analyzed were included in Table 1. When available, odor threshold in water [14,15,16,17,18,19,20,21] or aromatic notes were also obtained from the literature [14,15,22,23,24,25,26,27] (Table 1; Supplementary Table S1).

## 3. Results and Discussion

**Figure 1:**
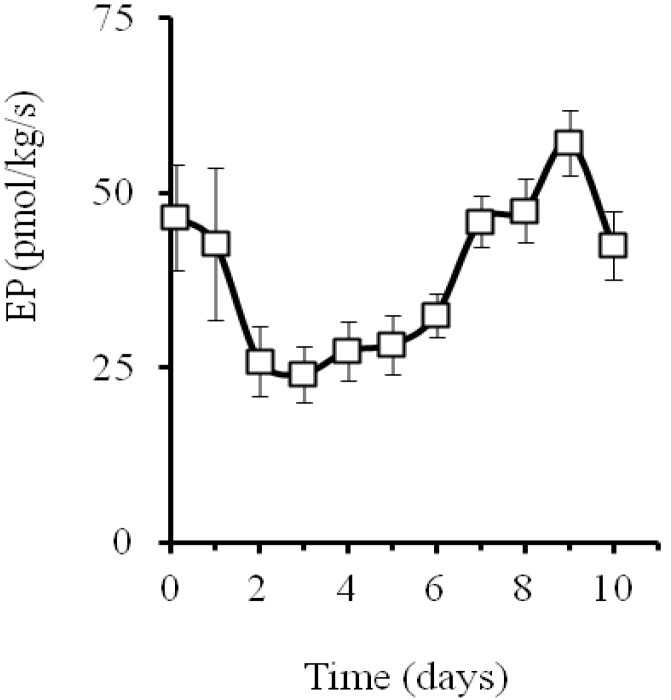
Ethylene production (EP) during ripening at 21 ± 1 °C and 93% ± 3% relative humidity of intact fruit of near-isogenic line SC3-5-1 (mean ± SE, *n* = 4).

The NIL SC3-5-1 showed a climacteric behavior from the 3rd day onwards, peaks on the 9th day with levels of 57.1 ± 2.5 pmol·kg^−1^·s^−1^ of ethylene, and decreased afterwards (Figure 1). This trend was accompanied by the typical climacteric levels of respiration rate with levels of 100–150 nmol·kg^−1^·s^−1^ (data not shown).

Twister technology is appropriate for monitoring changes in melon volatiles non-destructively because most of the well-known aromas in the climacteric melon NILs flesh were also recovered here [3,4]. The bars are small, easy to handle and manipulate in the laboratory, and can be easily transported after sampling in many different situations. Also, the bars can be stored for several months without volatile losses, are cheaper and do not have the risk of cracking compared with the SPME fibers. The disadvantage of PDMS bars is the lack of concentration of more polar compounds compared, for example, with Tenax^®^. This is the reason for the development of new bar coatings able to absorb compounds with different polarities, such as PDMS/polypyrrole, PDMS/metachrylate derivates, PDMS/activated carbon, PDMS-ACB, PDMS/polyvinylalcohol or PDMS/PVA, ethylene glycol (EG)-silicone; polyacrylate (PA) [28,29].

The aroma profile commonly found during ripening of NIL SC3-5-1 was mainly composed of 70 volatile compounds, 39 esters (18 acetate, 16 non-acetate, and five thioesters), seven organic acids, five aldehydes, four ketones, one alcohol, four terpenes, another three compounds of other chemical groups, and seven unidentified compounds (Table 1). Twenty one alkanes from C6 to C24 and C27 were identified but not included in the profile.

**Table 1:** Aroma volatile profile and concentration (average of relative area percentage on a total peak area basis) of compounds detected by headspace stir-bar sorptive extraction (1 h) during ripening at 21 ± 1 °C and 93% ± 3% relative humidity of intact fruit of near-isogenic line SC3-5-1 (mean ± SE, *n* = 4). Means ± SE during ripening or mean at harvest or at the climacteric (average of 8 and 10 day of measurement). NID, unidentified; IUPAC, international union of pure and applied chemistry; RT, retention time; CAS, Chemical Abstracts Service (NIST); LRI, linear retention index calculated from the RT of a series of straight-chain alkanes (C6–C20) or obtained from the literature; OT, odor threshold.

Profile id.	Compound (IUPAC name)	Time effect significant at *p* < 0.001	Aroma at harvest—or mean—(% area counts)	Aroma after 9 days or ± SE (% area counts)	RT (min) average	CAS number	LRI calculated average	LRI literature	Odor threshold in water (ppb)	Reference OT
	*Acetate esters*									
1	Ethyl acetate	NS	0.99±	0.2	1.911	000141-78-6	610	628	5–5000	[14]
2	Propyl acetate	*	1.71	1.22	2.357	000109-60-4	712	712	40	[15]
3	Butan-2-yl acetate	NS	0.39±	0.1	2.710	000105-46-4	750	766	3000	[15]
4	Butyl acetate	*	0.00	8.15	3.267	000123-86-4	810	806	66	[14]
5	3-Methylbutyl acetate	NS	1.72±	0.5	4.231	000123-92-2	873	875	3	[14,15]
6	2-Methylbutyl acetate	*	18.46	29.08	4.279	000624-41-9	876	880	2	[14]
7	2-Methylpropyl acetate	*	10.36	6.04	2.824	000110-19-0	764	768	66	[14]
8	Pentyl acetate	NS	1.03±	0.2	4.960	000628-63-7	912	909	7.5	[15]
9	Hexyl acetate	*	5.80	12.47	7.364	000142-92-7	1014	1010	2	[14]
10	Pent-4-Enyl acetate	NS	0.27±	0.0	4.396	001576-85-8	883	890	N/A	-
11	3-Methylbut-2-enyl acetate	NS	0.39±	0.1	5.180	001191-16-8	921	923	N/A	-
12	1-Acetyloxypropan-2-yl acetate	NS	0.07±	0.02	8.167	000623-84-7	1030	1036	N/A	-
13	3-Acetyloxybutan-2-yl acetate	NS	0.19±	0.02	9.307	001114-92-7	1063	1070	N/A	-
14	Heptyl acetate	NS	0.18±	0.03	11.163	000112-06-1	1115	1115	190	[16]
15	Benzyl acetate	*	19.34	6.21	13.276	000140-11-4	1167	1170	364	[17]
16	Octyl acetate	NS	0.36±	0.1	14.936	000112-14-1	1215	1212	12	[14]
17	2-Phenylethyl acetate	NS	1.82±	0.1	17.183	000103-45-7	1260	1264	480	[17]
18	[(1*R*,4*S*,6*R*)-1,7,7-Trimethyl-6-bicyclo[2.2.1]heptanyl] acetate	*	0.00	0.03	18.473	000125-12-2	1290	1290	N/A	-
	*Non-acetate esters*									
19	Ethyl butanoate	*	0.00	1.15	3.095	000105-54-4	797	800	1	[14]
20	Propyl propanoate	NS	0.56±	0.2	3.211	000106-36-5	806	807	57	[14]
21	2-Methylpropyl propanoate	*	1.49	0.85	4.068	000540-42-1	862	865	20	[18]
22	Propyl butanoate	NS	0.64±	0.1	4.632	000105-66-8	899	900	18	[14,18]
23	Butyl propanoate	NS	0.70±	0.1	4.839	000590-01-2	908	910	25	[14]
24	Propyl 2-methylbutanoate	NS	0.33±	0.1	5.713	037064-20-3	943	944	N/A	-
25	2-Methylpropyl butanoate	NS	2.53±	0.3	5.937	000539-90-2	952	934	1.6	[18]
26	3-Methylbutyl propanoate	NS	0.30±	0.1	6.322	000105-68-0	967	969	19	[16]
27	1-Butanol, 2-methyl-, propanoate	NS	2.14±	0.2	6.375	002438-20-2	969	975.6	19	[19]
28	2-Methylpropyl 2-methylbutanoate	NS	1.14±	0.1	7.284	002445-67-2	1004	1009	60	[16]
29	2-Methylbutyl 2-methylpropanoate	NS	0.56±	0.1	7.702	002445-69-4	1017	1015	N/A	-
30	Butyl 2-methylbutanoate	NS	0.24±	0.03	8.403	015706-73-7	1041	1048	61	[16]
31	3-Methylbutyl 2-methylpropanoate	*	0.58	0.03	9.038	002050-01-3	1056	1021	190	[16]
32	3-Methylbutyl butanoate	*	3.10	3.56	9.127	000106-27-4	1058	1043	N/A	-
33	2-Methylpropyl hexanoate	*	0.99	0.21	12.688	000105-79-3	1153	1149	N/A	-
34	3-Methylbutyl hexanoate	NS	0.19±	0.02	16.999	002198-61-0	1256	1254	N/A	-
	*Sulfur-derived compounds*									
35	1-Methylsulfanylethanone	*	1.37	0.66	2.270	001534-08-3	693	701	N/A	-
36	2-Methyl-2-(methylsulfanyl)butane	*	0.81	0.94	3.777	013286-92-5	843	842	N/A	-
37	1-Methylsulfanylbutan-1-one	*	0.48	0.84	4.385	002432-51-1	887	870	N/A	-
38	3-Methyl-1-methylsulfanyl-butan-1-one	*	2.28	9.25	5.565	023747-45-7	937	938	N/A	-
39	3-Methylsulfanylprop-1-ene	NS	0.54±	0.03	11.626	010152-76-8	1126	1133	0.14	[18]
	*Organic acids*									
40	Acetic acid	NS	0.94±	0.3	2.161	000064-19-7	667	622	22,000	[20]
41	Propanoic acid	*	0.36	0.09	2.490	000079-09-4	723	721	20,000	[14]
42	Hexanoic acid	NS	0.37±	0.1	6.901	000142-62-1	983	983	3000	[14]
43	Heptanoic acid	*	0.21	0.07	9.549	000111-14-8	1080	1078	3000	[14]
44	Octanoic Acid	*	0.57	0.16	13.773	000124-07-2	1179	1179	3000	[14]
45	Nonanoic acid	*	0.57	0.09	17.898	000112-05-0	1276	1278	3000	[14]
46	Hexadecanoic acid	*	4.31	0.05	44.889	000057-10-3	1967	1975	10,000	[14]
	*Aldehydes*									
47	Acetaldehyde	*	1.15	0.33	1.566	000075-07-0	-	500	15	[14]
48	Furan-2-carbaldehyde	*	0.82	0.21	3.620	000098-01-1	833	852	3000	[14]
49	Heptanal	*	0.28	0.05	4.709	000111-71-7	902	906	3	[14]
50	Benzaldehyde	*	1.58	0.29	6.144	000100-52-7	960	953	350	[14]
51	Decanal	NS	0.36±	0.1	14.955	000112-31-2	1208	1209	0.1	[14]
	*Ketones*									
52	Acetone	*	1.37	0.94	1.669	000067-64-1	610	503	500,000	[14]
53	4-Hydroxy-4-methylpentan-2-one	*	1.41	0.81	3.909	000123-42-2	852	846	270	[15]
54	6-Methylhept-5-en-2-one	NS	0.27±	0.1	6.856	000110-93-0	991	991	50	[14]
55	1-Phenylethanone	*	0.28	0.09	9.474	000098-86-2	1068	1065	65	[14]
	*Terpenes*									
56	4,7,7-Trimethylbicyclo[3.1.1]hept-3-ene	NS	0.29±	0.1	5.420	000080-56-8	931	933	6	[14]
57	1-Methyl-4-prop-1-en-2-yl-cyclohexene	*	0.17	0.08	8.087	005989-27-5	1028	1030	10	[14]
58	1,8,8-Trimethyl-7-oxabicyclo[2-2-2]octane	*	0.34	0.12	8.243	000470-82-6	1032	1030	12	[14]
59	α-(1*R*,2*S*,6*S*,7*S*,8*S*)-8-Isopropyl-1,3-dimethyltricyclo[4.4.0.0]dec-3-ene	*	0.14	0.12	22.243	003856-25-5	1377	1391	N/A	-
60	(3*E*,6*E*)-3,7,11-Trimethyldodeca-1,3,6,10-tetraene	NS	0.14±	0.03	27.878	000502-61-4	1505	1504	87	[21]
	*Other compounds*									
61	NID1	NS	0.52±	0.1	2.058	-	643	-	-	-
62	NID2	*	1.70	0.43	2.427		716	-	-	-
63	Methylbenzene	*	1.56	0.30	2.773	000108-88-3	758	773	330	[18]
64	NID3	NS	0.22±	0.1	10.723	-	1104	-	-	-
65	NID4	NS	0.27±	0.1	12.261	-	1104	-	-	-
66	Naphthalene	*	0.45	0.04	13.925	000091-20-3	1183	1179	9.5	[15]
67	NID5	NS	0.32±	0.10	26.687	-	1483	-	-	-
68	NID6	NS	0.46±	0.10	26.807	-	1485	-	-	-
69	NID7	NS	0.11±	0.03	26.861	-	1488	-	-	-
70	(*Z*)-Heptadec-8-ene	NS	0.76±	0.2	34.491	054290-12-9	1681	1679	N/A	-

**Figure 2:**
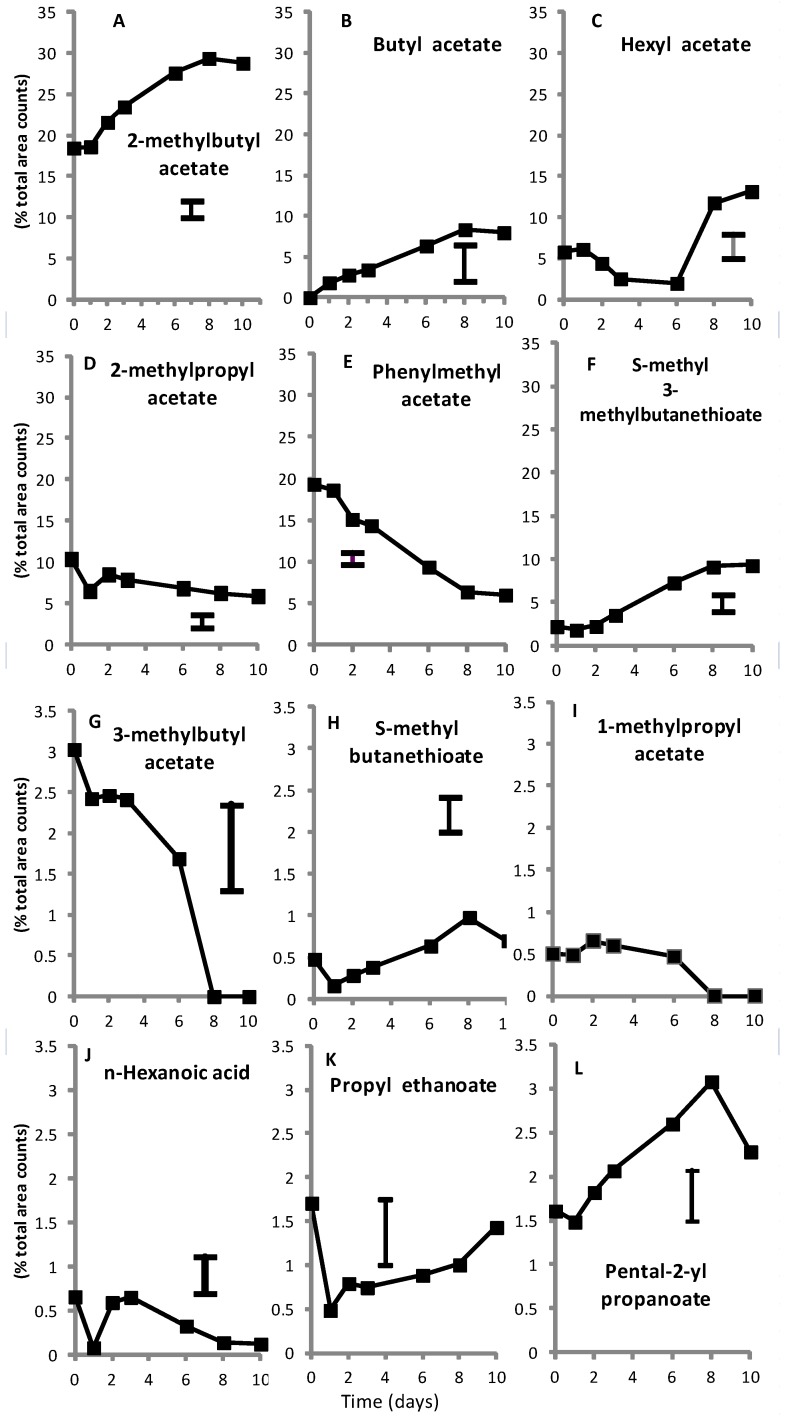
Relative concentration of individual aroma volatiles expressed as mean percentage of total area counts of the compounds identified per chromatogram and obtained by headspace stir-bar sorptive extraction (1 h) during ripening at 21 ± 1 °C and 93% ± 3% relative humidity of intact fruit of the near-isogenic line SC3-5-1 (mean ± SE, *n* = 4).

The profile was composed mainly of acetate and non-acetate esters (Figure 2A,C–E) with well-known odor descriptors (fruity, floral, *etc.*) (Supplementary Table S1). Some compounds, such as 3-methylbutyl propanoate of fruity odor, did not show significant changes over time. The most abundant compounds in SC3-5-1 that also increased during ripening were 2-methylbutyl acetate, representing 30% of the total area counts at the climacteric peak after 9 day (Figure 2A), followed by phenylmethyl acetate (Figure 2E) and others ranging from 0% to 2%, such as 2-methylpropyl acetate or *S*-methyl 3-methylbutanethioate (Figure 2). The 2-methylbutyl acetate is an odorant with intermediate intensity with an odor threshold value of 2 ppb in water (Supplementary Table S1). It has also been identified in “Jiashi” melon [30], and its amino acid precursor is l-isoleucine [31]. This compound is very abundant in Cantaloupe and “Charentais”-type melons [32] and is predominant together with butyl acetate and hexyl acetate in Galia-type melons [33]. Other non-acetate esters, such as pentan-2-yl propanoate (Figure 2L), also peaked at the climacteric peak.

The large amount of esters is also consistent with the strong dependence on ethylene biosynthesis of most of the ester and thioesters catalyzed by several alcohol acetyl transferases [34,35,36] and with methionine or other amino acids being precursors [31,37]. In fact, the pattern of many volatile compounds (e.g., Figure 2A–C,F,H,L) was concomitant with the upsurge in ethylene production (Figure 1), sometimes having important aromatic values at harvest (Figure 2A). In contrast, other volatiles such as phenylmethyl acetate, 3-methylbutyl acetate, or 1-methylpropyl acetate, decreased when the ethylene production increased (Figure 2E,G,I).

The volatile compounds were classified according to their pattern during postharvest ripening time. For example, four acetate esters (2-methylpropyl acetate; phenylmethyl acetate; 3-methylbutyl acetate and 1-methylpropyl acetate) decreased during ripening (Figure 2D,E,G,I). However, some thioesters (*S*-methyl 3-methylbutanethioate or *S*-methyl butanethioate; Figure 2F,H, respectively), 2-methyl-2-methylsulfanylbutane (from 0.8% to 1.2% after the 6th day), or non-acetate esters (*i.e.*, ethyl butanoate from 0% to 1.4% after 10 day of ripening), among other compounds, followed the opposite pattern. The data confirmed that all these compounds can be either detected in climacteric NILs either non-destructively (whole intact fruit, Table 1) or destructively (in the flesh) [3,4,13], and most of them are not apparently artefacts.

A few compounds rapidly declined after a relative maximum attained at harvest and were classified as typical harvest aroma compounds, such 2-methylpropyl hexanoate (from 1% to 0.2%–0.5% after 1–2 days of ripening), or *n*-hexadecanoic acid (from 4.3% to levels below 0.3% after 1 day of ripening). Other compounds decreased slowly during ripening, such a nonanoic acid (from 0.6% to less than 0.2% after 3 days of ripening) or *n*-hexanoic acid (Figure 2J). Probably the fast decline in some volatiles was particularly associated with melon plant detachment. In general, the decline of some compound may be considered as indication of its role as intermediate-acting compounds for the biosynthesis of others.

Finally, other volatiles were typical of melon senescence [4], though in some cases with similar levels at harvest and very close to the maximum ethylene peak, such as propyl ethanoate (Figure 2K). These compounds can be considered good candidates for validating optimum ripeness or for selecting fruit for immediate consumption or processing.

## 4. Conclusions

The acetate esters and thioesters, particularly 2-methylbutyl acetate, predominated in the SC3-5-1 profile. Aroma volatiles identified during ripening of the climacteric NIL SC3-5-1 followed different patterns but apparently following an ethylene-dependent pattern due to their biosynthesis or degradation.

## Supplementary Materials

Supplementary File 1
